# Self-Expandable Transcatheter Aortic Valve Implantation Outcomes: Findings From the Western Region of Saudi Arabia

**DOI:** 10.7759/cureus.18272

**Published:** 2021-09-25

**Authors:** Saad Albugami, Faisal Al-Husayni, Mawaddah Alfouti, Mohammed Algahtani, Wasna Alansari, Maha Hanawi, Ammar Albokhari, Wail Alkashkari, Hani N Mufti, Ahmed Krimly, Wail Alameen, Rasha Albawardy, Abdullah Alsaiedi

**Affiliations:** 1 Cardiology, King Abdullah International Medical Research Center, King Saud Bin Abdulaziz University for Health Sciences, Jeddah, SAU; 2 Cardiology, King Faisal Cardiac Center, King Abdulaziz Medical City, Ministry of National Guard Health Affairs, Jeddah, SAU; 3 Internal Medicine, King Abdullah International Medical Research Center, Jeddah, SAU; 4 Internal Medicine, National Guard Hospital, King Abdulaziz Medical City, Jeddah, SAU; 5 Interventional Cardiology, National Guard Hospital, Riyadh, SAU; 6 College of Medicine, King Saud Bin Abdulaziz University for Health Sciences, Jeddah, SAU; 7 Internal Medicine, Hera General Hospital, Makkah, SAU; 8 Medicine, King Abdullah International Medical Research Center, Jeddah, SAU; 9 Cardiac Surgery, King Faisal Cardiac Center, King Abdullah Medical City, Jeddah, SAU; 10 Medicine, King Saud Bin Abdulaziz University for Health Sciences, Jeddah, SAU

**Keywords:** aortic valve disease, transcatheter aortic valve implantation, tavi, aortic stenosis, tavr, transcatheter aortic replacement, outcomes

## Abstract

Background and objective

The prevalence of aortic stenosis in Saudi Arabia is expected to increase owing to the rise in the life expectancy of the population. Such increase is expected to be met with higher demand for interventions including transcatheter aortic valve implantation (TAVI). In this study, we aimed to identify the outcomes of this procedure among the population of the Western region of Saudi Arabia.

Methods

This was a retrospective observational study involving patients who underwent TAVI at the King Faisal Cardiac Center (KFCC), Jeddah, Saudi Arabia from June 2018 to January 2020. All patients who had undergone TAVI were included, and patients who were lost to follow-up for more than 90 days were excluded. The collected data included sociodemographic characteristics, peri-procedural assessment, and outcomes within 90 days.

Results

The study included a total of 52 patients. There were 28 males (53.8%) and 24 females (46.2%). The mean age of the cohort was 78 years. Type two diabetes mellitus was present in 67.3%, and hypertension and dyslipidemia were seen in 80.8% of patients. Coronary diseases were seen in 55.8%. The majority had prior percutaneous coronary intervention (PCI) (53.8%) and 3.8% had coronary artery bypass grafting (CABG). Twenty patients (38.5%) had heart failure. Atrial fibrillation was encountered in 13 patients (25%). Chronic kidney disease was described in nine (17.3%) patients, and four (7.7%) were on regular hemodialysis. The median Society of Thoracic Surgery (STS) risk score was 2.4 (IQR: 1.97-5). High STS scores (>8) were only seen in 9% of the patients. The success rate was 98%, and the in-hospital mortality rate was 3.8%. Vascular complications were seen in eight patients (15.4%), and the majority of them were minor. One patient (1.9%) had a major vascular complication. There was a tendency toward high blood transfusions (19.2%). Clinically manifest stroke was seen in three patients (5.8%). Eight patients (15.4%) had post-procedure complete heart block (CHB). Endocarditis was seen in two patients (3.8%). Thirty-day cardiac readmission was observed in 17.3% of patients, and acute kidney injury was seen in eight patients (15.4%). Mild aortic regurgitation was seen in 51.9% of the patients, but moderate or severe aortic insufficiency (AI) was not encountered.

Conclusion

Transfemoral TAVI using a self-expandable valve is a safe and feasible procedure at KFCC, an intermediate-sized center. Our data is comparable to local and international centers of similar size. Program sustainability depends on the development of robust referral networks and implementing regulatory quality and patient safety standards.

## Introduction

Transcatheter aortic valve implantation (TAVI) is now considered standard alternative therapy for the treatment of inoperable, high-risk, and intermediate-risk patients with severe symptomatic degenerative aortic valve stenosis (AS); moreover, recent study findings have expanded its scope to include patients with low surgical risk, with excellent short-term results [1-10]. Growing experience, device iterations, and technical procedural simplification have contributed to the widespread use of this modality, especially in developed and rich societies. In light of these considerations, many cardiac centers in Saudi Arabia have started to perform TAVI procedures. The fact that TAVI is a less invasive procedure compared to surgical aortic valve replacement (SAVR) makes it extremely attractive for many patients, and hence many of them prefer it over SAVR, particularly since the cost is borne by the government and medical services are free. Despite the early introduction of TAVI in Saudi Arabia, as far back as 2007, there is scarce data on its safety [11,12]. The published reports, though limited, have shown favorable outcomes with low rates of complications and mortality with first-generation devices. In this report, we sought to study the outcomes of TAVI using self-expandable second-generation devices (Medtronic CoreValve™ or Evolut™ R valves; Medtronic, Minneapolis, MN) at an intermediate volume center: the King Faisal Cardiac Center (KFCC) in Jeddah, Saudi Arabia.

## Materials and methods

This was a single-center, retrospective, and descriptive study of all consecutive patients who underwent TAVI at KFCC, Jeddah, Saudi Arabia from June 2018 till January 2020. Patients were considered suitable candidates if they had severe symptomatic native AS and were at a high or prohibitive surgical risk, or had been declined cardiac surgery by two experienced cardiac surgeons during the heart team discussion. Severe AS was defined as an aortic valve area of <1 cm^2^ or <0.6 cm^2^/m^2^, a mean aortic-valve pressure gradient of 40 mmHg, or a peak aortic-jet velocity of P 4.0 m/s, as measured by echocardiography, or patients with low-flow, low-gradient symptomatic AS with demonstrated high aortic valve leaflet calcification scores. The high or prohibitive surgical risk was defined as patients being above the age of 80 years or on hemodialysis or having an STS risk score of 8 or above with a life expectancy of more than 12 months. Data were collected for all patients who had undergone TAVI. Before employing the procedure, all patients were reviewed by the heart team and informed consent was obtained. Data included sociodemographic information, past medical and surgical history, symptoms, and laboratory values (routine blood work, cardiac markers). In-hospital outcomes, electrocardiogram (ECG), and echocardiogram (Echo) findings were also collected. Ninety-day outcomes were also measured, which included TAVI-related complications, emergency room visits, and TAVI-related readmission rates. The study received ethical approval from the Institutional Review Board of the King Abdullah International Medical Research Center.

Statistical analysis

Statistical analysis was conducted using the R software version 4.0.2 (R Foundation For Statistical Computing, Vienna, Austria). We analyzed the general characteristics of all patients (n=52) who had undergone TAVI at our institution. Because of the small sample size, we relied on nonparametric measures of central tendency and dispersion. For continuous variables, the median and interquartile range were used. The Wilcoxon rank-sum test and Kruskal-Wallis test were used for comparison between groups. Absolute and relative frequencies were used for categorical variables and analyzed using either the Chi-squared or the Fisher’s exact test. All statistical tests were two-tailed, and a p-value of <0.05 was considered statistically significant.

## Results

The study included all patients who underwent TAVI with self-expandable aortic valve prosthesis (Medtronic CoreValve™ or Evolut™ R valves; Medtronic, Minneapolis, MN) at KFCC from June 2018 till January 2020. There were only 52 patients; 28 were males (53.8%) and 24 were females (46.2%). The mean age of the cohort was 78 years. Risk factors were prevalent among this group of patients. Type two diabetes mellitus was present in 67.3% of the patients; hypertension and dyslipidemia were seen in 80.8%, and coronary disease was seen in 55.8%. The majority had prior percutaneous coronary intervention (PCI) (53.8%) and 3.8% had coronary artery bypass grafting (CABG). Twenty patients (38.5%) had heart failure; atrial fibrillation was encountered in 13 patients (25%). Chronic kidney disease was described in nine (17.3%) patients, and four of them (7.7%) were on regular hemodialysis (Table 1).

**Table 1:** Patients’ general characteristics IQR: interquartile range; DM: diabetes mellitus; HTN: hypertension; DLP: dyslipidemia; COPD: chronic obstructive pulmonary disease; HF: heart failure; A.Fib: atrial fibrillation; CAD: coronary artery disease; CKD: chronic kidney disease; PVD: peripheral vascular disease; PCI: percutaneous coronary intervention; CABG: coronary artery bypass grafting

Patient characteristics	All patients (n=52)
Age in years, mean (IQR); male, female, n (%)	78 (71-83); 28 (53.8%), 24 (46.2%)
Smoking, n (%)	4 (7.7)
DM, n (%)	35 (67.3)
HTN, n (%)	42 (80.8)
DLP, n (%)	42 (80.8)
COPD, n (%)	2 (3.8)
Asthma, n (%)	4 (7.7)
HF, n (%)	20 (38.5)
A.Fib, n (%)	13 (25)
Stroke, n (%)	9 (17.3)
CAD, n (%)	29 (55.8)
CKD, n (%)	9 (17.3)
Hemodialysis, n (%)	4 (7.7)
PVD, n (%)	4 (7.7)
PCI, n (%)	28 (53.8)
CABG, n (%)	2 (3.8)
Valve surgery, n (%)	1 (1.9)

The presenting symptoms are described in Table 2. The predominant symptoms were chest pain (26.9%) and dizziness (26.9%). Syncope was less frequent (13.5%).

**Table 2:** Pre-TAVI symptoms TAVI: transcatheter aortic valve implantation

Symptoms	All patients (n=52)
Chest pain, n (%)	14 (26.9)
Dizziness, n (%)	14 (26.9)
Palpitation, n (%)	2 (3.8)
Syncope, n (%)	7 (13.5)
Cough, n (%)	3 (5.8)

The median Society of Thoracic Surgery (STS) risk score was 2.4 (IQR: 1.97-5). High STS scores (>8) were only seen in 9% of the patients; the rest had intermediate to low risk based on the STS calculator, but those patients were deemed high risk by the heart team members due to age, comorbidities, and frailty (Table 3).

**Table 3:** Pre-TAVI data TAVI: transcatheter aortic valve implantation; IQR: interquartile range; SBP: systolic blood pressure; DBP: diastolic blood pressure; HR: heart rate; O_2_ Sat: oxygen saturation; BNP: B-type natriuretic peptide; TropI: troponin; RV: right ventricle; AS: aortic valve stenosis; RVSP: right ventricular systolic pressure; AV: aortic valve, STS: the Society of Thoracic Surgeons

Patient characteristics	All patients (n=52)
SBP, mmHg, median (IQR)	126 (112-139)
DBP, mmHg, median (IQR)	60 (55-66)
HR, bpm, median (IQR)	73 (66-82)
O_2_ Sat, %, median (IQR)	98 (96-99)
Hemoglobin, gm/dL, median (IQR)	12.2 (10.4-13.2)
Platelets, x 10^9^/L, median (IQR)	230 (195-285)
Creatinine, μmol/L, median (IQR)	86 (69-112)
BNP, pg/mL, median (IQR)	236 (145-476)
TropI, pg/mL, median (IQR)	19.7 (13-245)
RV dysfunction, n (%)	2 (3.9)
Severe AS, n (%)	49 (94.2)
RVSP, mmHg, median (IQR)	17 (9-26)
AV mean gradient on Echo, mmHg, median (IQR)	39.8 (28.1-53.5)
STS mortality score, median (IQR)	2.44 (1.97-5)

Echocardiographic parameters were consistent with severe AS with a mean AVA of 1 cm^2^ and median aortic valve gradient of 39.8 mmHg (28.1-53.5 mmHg). Post-TAVI, there was an improvement in these parameters: mean AVA increased to 1.8 cm^2^, and mean gradient decreased to 6.0 mmHg. Mild aortic regurgitation degree post-TAVI was seen in 51.9%, and none were moderate or severe (Table 4).

**Table 4:** Aortic regurgitation pre- and post-TAVI AR: aortic regurgitation; TAVI: transcatheter aortic valve implantation

AR	Pre-TAVI, n (%)	Post-TAVI, n (%)
None	11 (21.2)	25 (48.1)
Mild	24 (46.2)	27 (51.9)
Moderate	14 (26.9)	0
Severe	3 (5.8)	0

Table 5 shows the patient outcomes after TAVI. There were no significant changes in the left ventricular ejection fraction. The success rate was 98%, and the in-hospital mortality rate was 3.8% (two patients died due to heart failure complications and cardiogenic shock). One patient died in the first 30 days following discharge due to pneumonia (1.9%). Vascular complications were seen in eight patients (15.4%), and the majority of them were minor. One patient had a major vascular complication that required surgical cut-down due to femoral artery dissection and occlusion following closure device deployment (1.9%). There was a tendency toward high blood transfusion (19.2%). Clinically manifest stroke was seen in three patients (5.8%). Eight patients (15.4%) had post-procedure complete heart block (CHB) that required a permanent pacemaker within 48 hours. The latter 18 patients' permanent pacemaker rate was zero due to the adoption of cusps overlap views during device deployment. Endocarditis was seen in two patients (3.8%), both treated medically with antibiotics; one completely recovered but the other one had a stroke but survived. The 30-day cardiac readmission rate was 17.3%. Acute kidney injury was seen in eight patients (15.4%), and all of them were managed conservatively.

**Table 5:** Outcomes post-TAVI IQR: interquartile range; MI: myocardial infarction; ER: emergency room; AR: aortic valve regurgitation; AV: aortic valve; TAVI: transcatheter aortic valve implantation

Patient characteristics	All patients (n=52)
Length of hospital stay, days, median (IQR)	7 (4-11)
In-hospital mortality, n (%)	2 (3.8)
Mortality after discharge, n (%)	1 (1.9)
Acute kidney injury, n (%)	8 (15.4)
Blood transfusion, n (%)	10 (19.2)
Vascular complications, n (%)	8 (15.4)
Permanent pacemaker, n (%)	8 (15.4)
MI, n (%)	2 (3.8)
Stroke, n (%)	3 (5.8)
Endocarditis, n (%)	2 (3.8)
Cardiac-related ER visit, n (%)	14 (26.9)
Cardiac-related readmission, n (%)	9 (17.3)
Mild AR, n (%)	27 (51.9)
AV mean gradient post-TAVI, mmHg, median (IQR)	7.8 (5.6-11)

## Discussion

TAVI is now a routine procedure in many centers in Saudi Arabia. The expanding clinical indications and the availability of the technology have made it possible for many small and intermediate centers all over the country to commence their own programs. KFCC is an intermediate volume center that has established this vital service to its patients due to high demand; moreover, it has become a necessity to expand the clinical portfolio of many cardiac centers. Our study, albeit the small sample size, has demonstrated excellent outcomes with TAVI by using an expandable aortic valve prosthesis for patients with severe symptomatic AS in an intermediate-sized center. The study confirms what was reported by Alatawi et al. [13], a similarly sized study that showed favorable results of the procedure. Our patients had a lower STS risk score when compared to local and international studies although they were found to be at high risk by the heart team members.

All procedures were done through the femoral access using the available self-expandable prosthesis. TAVI delivery was successful in all except one patient in whom we were unable to cross the native aortic valve due to extreme aneurysmal dilatation of the aortic root (attempted valve in valve). The procedural complications were comparable to what was published previously in local and international studies [13,14]. The in-hospital mortality of 3.8% was in line with that of other local centers [13]. The reported in-hospital mortality from German registries is inversely related to the hospital case volume as reported by Bestehorn et al. [15] who reported an average in-hospital mortality of 5.6 ±5.0% (range: 0-16.7%) in the lowest volume group of hospitals performing <50 transfemoral (TF)-TAVI annually, compared to 2.4 ±1.0% (range: 0.5-3.7%) in the highest volume hospitals with ≥200 TF-TAVI procedures per year. Vascular complications were seen in 15.4% of the patients, and the majority of them were minor. There was a tendency toward a high prevalence of blood transfusion (19.2%). Clinically manifest stroke was seen in 5.8%, which was slightly higher than the 5% that was reported from early TAVI trials [14]. CHB during or post-procedure was encountered in 15.4% of the patients. Following the implementation of cusp overlap views [15] during deployment, in addition to enhancing experience, the rate of CHB in the latter 18 cases came down to zero.

Acute kidney injury was encountered in 15.4% of the cases, which is consistent with what was reported in other local studies (17.3%), and, similar to what was reported by Alatawi et al. [13], none of those patients required new renal replacement therapy. The rate of mild and trivial paravalvular leak was 51.9%, while non-developed moderate or severe paravalvular leak required intervention during the follow-up.

Our study, although small-sized, confirms and validates the benefits of TAVI in small and intermediate volume programs. However, measures for quality improvement are still required as high-volume programs attain better outcomes, as shown by Desai et al. who have reported substantial variations in the quality of transcatheter aortic valve replacement (TAVR) care received in the United States; 11% of the sites were identified as providing care below the average level of performance. Only 8% of sites performed better than expected, and most sites (80%) lived up to the expectation in terms of performance [16]. Other factors contributing to below-average outcomes have not yet been published.

Our report describes the first 52 cases of TAVI using a single device at our center, and it shows a clear learning curve effect when comparing the early versus late complications, especially regarding the rate of permanent pacemaker incidence. The development of TAVI centers is important in a big and vast country like Saudi Arabia, but it is of paramount importance to focus on the development of strong referral networks to improve patient outcomes while ensuring adequate access to care [17].

Limitations

Our study was retrospective and observational in nature, and hence it has an inherent selection bias. In addition, it was a single-center study, which may affect the generalizability of its conclusions. Our assessment of TAVI was limited to self-expandable prostheses only, and we did not include balloon-expandable ones. Furthermore, the assessment of the functional improvement was not made. Another limitation is the small number of patients included in the study and the lack of more than 90-day outcomes. However, despite these limitations, the results we obtained reflect data consistent with other local outcome studies regarding TAVI.

## Conclusions

TAVI using a self-expandable device is a safe and feasible procedure and is associated with favorable outcomes in intermediate-sized centers. Continuous development of sustainable programs and networks for referrals will translate into further improvement in care and patient outcomes in general.
